# Redox Proteomics of the Inflammatory Secretome Identifies a Common Set of Redoxins and Other Glutathionylated Proteins Released in Inflammation, Influenza Virus Infection and Oxidative Stress

**DOI:** 10.1371/journal.pone.0127086

**Published:** 2015-05-18

**Authors:** Paola Checconi, Sonia Salzano, Lucas Bowler, Lisa Mullen, Manuela Mengozzi, Eva-Maria Hanschmann, Christopher Horst Lillig, Rossella Sgarbanti, Simona Panella, Lucia Nencioni, Anna Teresa Palamara, Pietro Ghezzi

**Affiliations:** 1 Institute Pasteur, Cenci-Bolognetti Foundation, "Sapienza" University of Rome, Rome, Italy; 2 Brighton & Sussex Medical School, Falmer, Brighton, United Kingdom; 3 University of Brighton, Pharmacy and Biomolecular Sciences, Moulsecoomb, Brighton, United Kingdom; 4 Institute for Medical Biochemistry and Molecular Biology, University Medicine, Ernst-Moritz Arndt University, Greifswald, Germany; 5 IRCSS San Raffaele Pisana, Telematic University, Rome, Italy; 6 Department of Public Health and Infectious Diseases, Institute Pasteur Cenci-Bolognetti Foundation, "Sapienza" University of Rome, Rome, Italy; University of Valencia, SPAIN

## Abstract

Protein cysteines can form transient disulfides with glutathione (GSH), resulting in the production of glutathionylated proteins, and this process is regarded as a mechanism by which the redox state of the cell can regulate protein function. Most studies on redox regulation of immunity have focused on intracellular proteins. In this study we have used redox proteomics to identify those proteins released in glutathionylated form by macrophages stimulated with lipopolysaccharide (LPS) after pre-loading the cells with biotinylated GSH. Of the several proteins identified in the redox secretome, we have selected a number for validation. Proteomic analysis indicated that LPS stimulated the release of peroxiredoxin (PRDX) 1, PRDX2, vimentin (VIM), profilin1 (PFN1) and thioredoxin 1 (TXN1). For PRDX1 and TXN1, we were able to confirm that the released protein is glutathionylated. PRDX1, PRDX2 and TXN1 were also released by the human pulmonary epithelial cell line, A549, infected with influenza virus. The release of the proteins identified was inhibited by the anti-inflammatory glucocorticoid, dexamethasone (DEX), which also inhibited tumor necrosis factor (TNF)-α release, and by thiol antioxidants (N-butanoyl GSH derivative, GSH-C4, and N-acetylcysteine (NAC), which did not affect TNF-α production. The proteins identified could be useful as biomarkers of oxidative stress associated with inflammation, and further studies will be required to investigate if the extracellular forms of these proteins has immunoregulatory functions.

## INTRODUCTION

Infection, autoimmunity, tissue stress and tissue injury can all induce inflammation [1]. Pathogens, through specific pathogen-associated molecular patterns such as endotoxin, viral proteins or nucleic acids, induce expression and release of inflammatory cytokines through activation of various pattern recognition receptors including Toll-like receptors [2, 3]. They also induce the release of endogenous proteins that are normally present intracellularly such as high-mobility group box-1 (HMGB1) [4]. These proteins are often classified as damage-associated molecular patterns because, being normally present in the cell, are obviously also released as a result of necrosis, independent of the mechanism that triggered cell death [5].

Oxidative stress is caused by an imbalance between the production of reactive oxygen species (ROS) and the ROS-detoxifying capacity of the cells [6]. ROS are thought to play a role in triggering or sustaining the inflammatory response, and may be particularly important in pathological conditions such as ischemia/reperfusion injury, typically associated with high oxidative stress [7], but also in infectious diseases such as influenza [8–10] or during HIV infection [11, 12]. Pioneering studies by Bauerle have shown that nuclear factor-kappa B is one of the redox-sensitive targets in inflammation, as shown by its activation by ROS and its inhibition by thiol antioxidants such as glutathione (GSH) [13]. More recently, other signalling molecules in the inflammatory cascade, including a member of the NLR family, the pyrin-like protein, NALP3 (a signal transducer and activator of transcription), have been shown to be redox regulated [14, 15]. On the other hand, while a number of studies reported inhibition of cytokine production by thiol antioxidants, possibly through the above-mentioned inhibition of nuclear factor-kappa B, it is unclear whether ROS alone can directly trigger inflammatory cytokines and to what extent. It has been reported that low levels of hydrogen peroxide induces production of IL-1, TNF, and chemokines in mouse peritoneal macrophages [16], and we previously reported that a ROS-generating toxicant, paraquat, potentiates induction of IL-1, IL-8 and TNF by LPS but does not induce these cytokines in the absence of LPS [17, 18]. In contrast, hydrogen peroxide, even at non-toxic concentrations, had a marked effect on the release of HMGB1, indicating that damage-associated molecular patterns might be an important mediator of oxidative stress-associated inflammation [19].

Recently, the focus has shifted from the concept of oxidative damage to that of redox regulation. According to the latter concept, there are a number of metabolic pathways that are regulated by the redox state of the cell, which is defined as the reduced/oxidized ratio for some metabolites, particularly NADH/NAD, NADPH/NADP, GSH/GSSG [20]. One mechanism by which the redox state of the cell affects the function of proteins, for instance, enzymes, transcription factors and transporters, is via oxidoreduction of redox-sensitive cysteines in the protein sequence. This can occur in a number of ways: reversible formation of disulfide bonds within the same protein or between distinct proteins; formation of mixed disulfides with small-molecular weight thiols including GSH (protein glutathionylation), or free cysteine (protein cysteinylation) [21]; or via other forms of reversible oxidation such as formation of S-nitrosothiols or other oxidized species, for instance sulfenic or sulfinic acids [22].

We previously developed “redox proteomics” techniques to identify intracellular proteins whose cysteines are redox-sensitive, and successfully applied it to the identification of cysteines undergoing glutathionylation in lymphocytes [23], or membrane proteins whose exofacial thiols are potential targets of redox regulation [24, 25]. Recently, we applied this technique to identify those proteins that are released in the glutathionylated form by LPS-stimulated macrophages, and identified PRDX2 as a potential inflammatory mediator [26].

We report here a list of other proteins identified as released in the glutathionylated form following LPS-stimulation of RAW264.7 mouse macrophages, a cell line which has been used for the identification of inflammatory mediators such as TNF [27] and HMGB1 [4]. The strategy employed was based on the use of a biotinylated GSH ethyl ester, (BioGEE) to label the intracellular GSH pool [28]. Glutathionylated proteins released in the LPS-stimulated supernatant would then carry a biotin tag and could be purified by binding to streptavidin-agarose followed by elution, tryptic digestion and identification by mass spectrometry. Release of specific proteins, augmentation of release by LPS and glutathionylation of the secreted protein were then confirmed using more specific techniques. [29]We also studied the effect of infection with influenza virus, known to induce a decrease of GSH in the cell [30, 31]. Finally we tested the effect of a classical anti-inflammatory agent, dexamethasone phosphate (DEX), and two thiol antioxidants, the GSH synthesis precursor N-acetyl-L-cysteine (NAC) [32] and the cell-permeable N-butanoyl GSH derivative, GSH-C4 [33, 34] on LPS-stimulated release of the proteins identified.

The results indicate that the LPS-induced release of a number of the proteins investigated can be pharmacologically modulated by thiol antioxidants and anti-inflammatory drugs.

## RESULTS

### Identification of redoxins and other glutathionylated proteins released in response to LPS stimulation

The protocol for the identification of glutathionylated proteins released by LPS-stimulated RAW264.7 mouse macrophages has been described before [26]. Briefly, supernatants from cells pre-loaded or not with BioGEE and then stimulated with LPS were run on a non-reducing SDS-PAGE followed by Western blot with streptavidin-peroxidase (S1 Fig). A number of proteins were detected in the releasate, while supernatants from cells without BioGEE treatment gave no visible bands. When samples were reduced with DTT before electrophoresis, fewer bands were stained by streptavidin-peroxidase, indicating that in most cases incorporation of biotin was due to a mixed disulfide formation, as it would be in the case of glutathionylation. Similar results were obtained when cells were pre-loaded with BioGEE for 1 h, then BioGEE was removed and the released biotinylated proteins analysed by Western blot (S1B Fig).

To identify BioGEE-labelled proteins we used two different strategies, which essentially differed in the use of prior electrophoretic separation. In the first experiment, proteins in the supernatant from BioGEE-preloaded, LPS-stimulated cells were affinity-purified on streptavidin beads, and bound proteins were identified following tryptic digestion *in situ* by LC-MS/MS. A parallel sample prepared without BioGEE was processed in the same way and proteins were also identified. The full list of proteins is available as Supporting Information, S1 Table. In the second experiment, the supernatant from BioGEE-preloaded, LPS-stimulated cells was incubated with streptavidin agarose, eluted with DTT for 30 min at room temperature and then again with DTT for 10 min at 100°C. The eluate was then fractionated by SDS-PAGE under reducing conditions and proteins visualised by staining with Coomassie Blue. The three visible bands (S2 Fig) were excised and subjected to tryptic digestion and MS analysis. The proteins identified are listed in the Supporting information, S2 Table.

Table 1 lists the proteins identified in the first experiment from BioGEE-preloaded, LPS-stimulated cells. The columns on the left indicate which proteins were unique to BioGEE-pretreated cells, as detection in the absence of BioGEE may indicate non-specific binding of the protein to the beads. The last column of Table 1 indicates those proteins that were also identified in excised gel slices from the SDS-PAGE separation. Although this might be a confirmation of the identification, one should bear in mind that only peptides from three bands were 'sequenced', thus limiting the identification to those proteins that are in a limited molecular weight range.

**Table 1:** Proteins identified from BioGEE-treated samples.

Protein name	Accession number	Unique	Gel
50 kDa protein	IPI00311569, IPI00462482	√	
78 kDa glucose-regulated protein	IPI00319992		√
Actin, cytoplasmic 1	IPI00110850		
Alpha-enolase	IPI00462072		
Bifunctional aminoacyl-tRNA synthetase	IPI00339916, IPI00673707	√	
Cathepsin B	IPI00113517	√	
Elongation factor 1-alpha 1	IPI00307837		
Elongation factor 1-gamma	IPI00318841		
Elongation factor 2	IPI00466069		√
Envelope polyprotein	IPI00420148, IPI00626977, IPI00754463, IPI00845606, IPI00845754, IPI00845857, IPI00923031	√	
Exportin-1	IPI00395038	√	
Fatty acid synthase	IPI00113223	√	
Glucose-6-phosphate 1-dehydrogenase X	IPI00228385, IPI00857114	√	
H-2 class I histocompatibility antigen, L-D alpha chain	IPI00109996	√	
Heat shock 70 kDa protein 4	IPI00331556	√	
Heat shock cognate 71 kDa protein	IPI00323357, IPI00886297		√
Heat shock protein HSP 90-alpha	IPI00330804		√
Hypoxanthine-guanine phosphoribosyltransferase	IPI00284806	√	
Isoform 1 of Filamin-A	IPI00131138, IPI00664643, IPI00875567, IPI00921658	√	
Isoform 1 of Interleukin-1 receptor antagonist protein	IPI00136858, IPI00466271, IPI00751888, IPI00752375		
Isoform 2 of Tropomyosin alpha-3 chain	IPI00230044, IPI00459570	√	
Isoform C of Lamin-A/C	IPI00400300, IPI00620256		
Isoform M2 of Pyruvate kinase isozymes M1/M2	IPI00407130, IPI00845840		
Keratin, type II cytoskeletal 1	IPI00625729		
L-lactate dehydrogenase A chain	IPI00319994, IPI00751369	√	
Multifunctional protein ADE2	IPI00322096, IPI00624863	√	
Nucleolin	IPI00317794		√
Peptidyl-prolyl cis-trans isomerase B	IPI00135686		
Peroxiredoxin-1	IPI00121788, IPI00648105, IPI00648615		
Peroxiredoxin-2	IPI00117910	√	
Phosphoglycerate kinase 1	IPI00555069		
Plastin-2	IPI00118892		
Profilin-1	IPI00224740, IPI00650039		√
Protein disulfide-isomerase A3	IPI00230108		
Putative uncharacterized protein	IPI00309520, IPI00877231		
Putative uncharacterized protein	IPI00229080		
Putative uncharacterized protein	IPI00126248, IPI00762047		
similar to Protein disulfide isomerase associated 6	IPI00854971		
Sulfated glycoprotein 1	IPI00321190, IPI00928070, IPI00928204, IPI00928284, IPI00928320, IPI00928581	√	
T-complex protein 1 subunit epsilon	IPI00116279	√	
Transgelin-2	IPI00125778		
Transketolase	IPI00137409	√	√
Triosephosphate isomerase	IPI00467833		
Tubulin alpha-1B chain	IPI00117348		
Tubulin beta-5 chain	IPI00117352		
Vimentin	IPI00227299	√	

**Fig 1:**
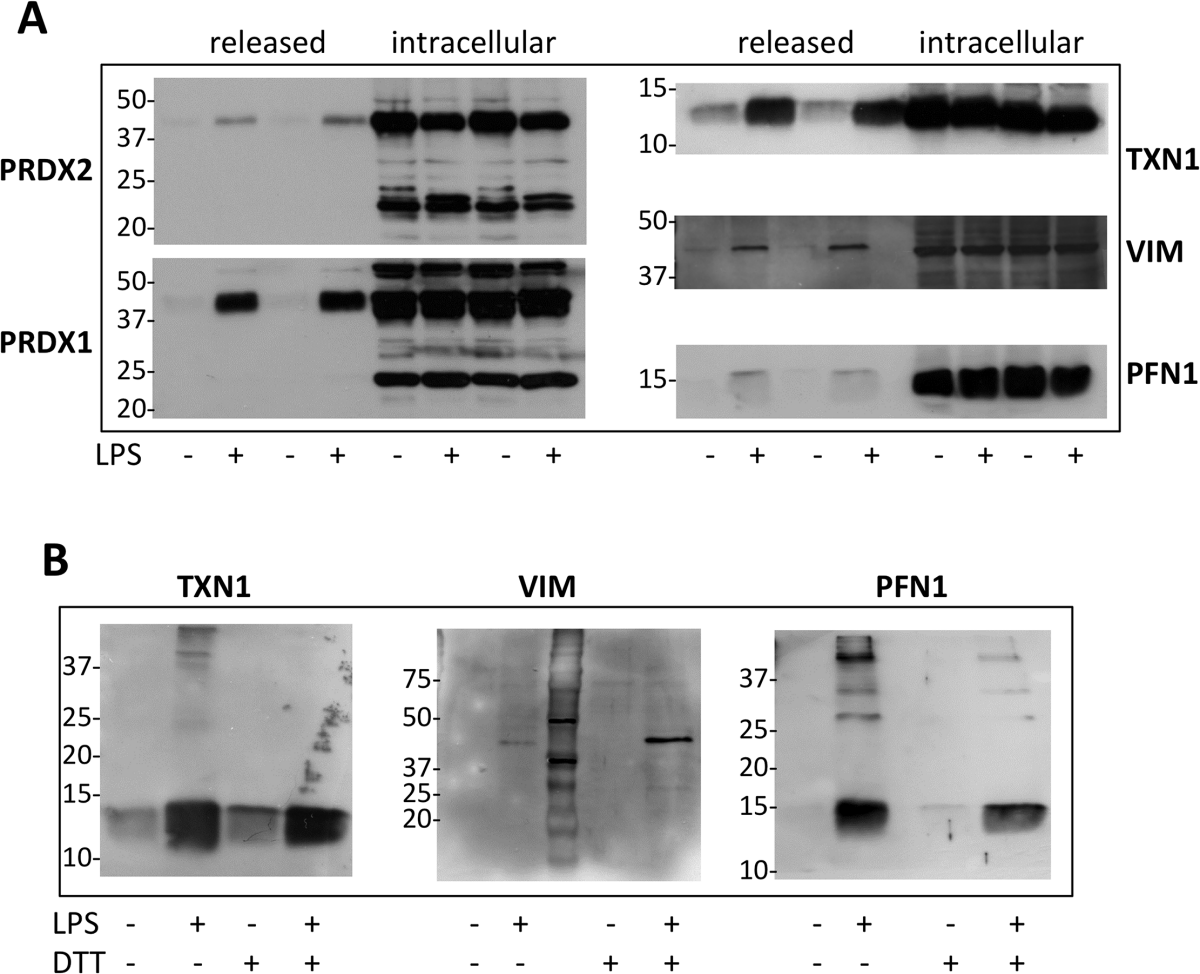
LPS induces release of PRDX1, PRDX2, TXN1, VIM and PFN1. Western blot analysis following non-reducing (A) or reducing (B) SDS-PAGE (10% acrylamide for VIM, 12% for PRDXs, 15% for TXN1 and PFN1) of RAW264.7 supernatants cultured with and without 100 ng/ml LPS for 24 h. Supernatants were blocked with 40 mM NEM immediately after collection, to prevent thiol-disulfide exchange. Cell lysates were also analyzed after blocking with NEM.

We decided to further investigate peroxiredoxin (PRDX) 1, PRDX2, vimentin (VIM) and profilin1 (PFN1), all identified in the first experiment, as well as thioredoxin 1 (TXN1), identified only in the second experiment. PRDX2 and TXN1 have already been reported as secreted by LPS-stimulated macrophages in our previous study [26]. We decided to investigate PFN1 because, although in this study it was also identified in the absence of BioGEE treatment, it had already been identified as susceptible to glutathionylation in a previous redox proteomics study, although in the cytosol [23]. Likewise, PRDX1 was selected for further study, even though it was also identified in the absence of BioGEE treatment because of its similarity to PRDX2. Because the mass spectrometry method used in this study for protein identification is not quantitative, and we only analyzed supernatants from LPS-stimulated cells, identification of a protein in our redox proteomics experiment does not necessarily imply the protein is released in response to LPS. Furthermore, proteins identified in this way may also be contaminants released from dead cells. Therefore, we first sought to confirm the release of these proteins by Western blotting using specific antibodies. Fig 1A shows Western blot analysis for the different proteins in supernatants and cell lysates 24 h after stimulation with or without 100 ng/ml of LPS. In this experiment, SDS-PAGE was run under non-reducing conditions. PRDX2 is present intracellularly as both a monomer and a disulphide-linked dimer, and LPS induces release of the dimer but not of the monomer, as previously reported [26]; we report here the same pattern of secretion for PRDX1.

We were also able to demonstrate that TXN1, VIM and PFN1 are constitutively present in the cell and released in response to LPS. Unlike PRDXs, these proteins ran only as a major band on the SDS-PAGE gels, migrating near their predicted molecular weight. Although in non-reducing conditions it is impossible to determine the accurate molecular weight as protein migration is also affected by other factors, such as 3D conformation and the presence of disulfide bonds, it was clear that these proteins were not released as dimers. The results with VIM are less clear. The molecular weight of mouse VIM is 53 kDa but in our Western blots the main band is at 42–44 kDa, both in supernatants and in the cell lysate (Fig 1A). We thus also analysed secreted TXN1, VIM and PFN1 in reducing conditions. As shown in Fig 1B, TXN1 and PFN1 migrate to the expected molecular weight (12 kDa and 15 kDa, respectively), but once again VIM showed a lower molecular weight than expected, and for this reason it was not investigated further in the present study.

We wondered whether the fact that secreted PRDXs are only found as dimers is not simply due to their oxidization after secretion in the oxidizing extracellular milieu that might also contain hydrogen peroxide released by the cells. However, when human recombinant, monomeric, PRDX2 was added to cultured cells, we could still detect the monomer after 24 h culture (S3 Fig) indicating that dimerization probably occurs before or during the process of release, rather than once it is released. This is in agreement with our previous findings in HEK293 cells [35].

Given the novelty of the findings, we wanted to exclude beyond any doubt the possibility that the release of the proteins investigated was not due to leakage associated with a small amount of cell lysis possibly induced by LPS. As a first approach, we measured cell viability using two different assays.

Using CellTiter Blue, viability of cells treated 24 h with 100 ng/ml LPS was 99.6 ± 1.9% of control cells (n = 4). As shown in S4 Fig, the assay used would have detected a 5% decrease in the number of live cells. Because the CellTiter Blue assay is based on an oxidoreductive reaction, we wanted to confirm these measurements with a different assay. We used the CellTiter Glo assay that is based on the measure of the ATP content of the cells. Also with this assay, viability of cells treated 24 h with 100 ng/ml LPS was 99.6 ± 7.0% of control cells (n = 10). Furthermore, we measured TNF production in the 24-h supernatants, and LPS induced high TNF levels (control, 0.7 ± 0.1 ng/ml; LPS, 99.2 ± 1.3 ng/ml; n = 3).

**Fig 2:**
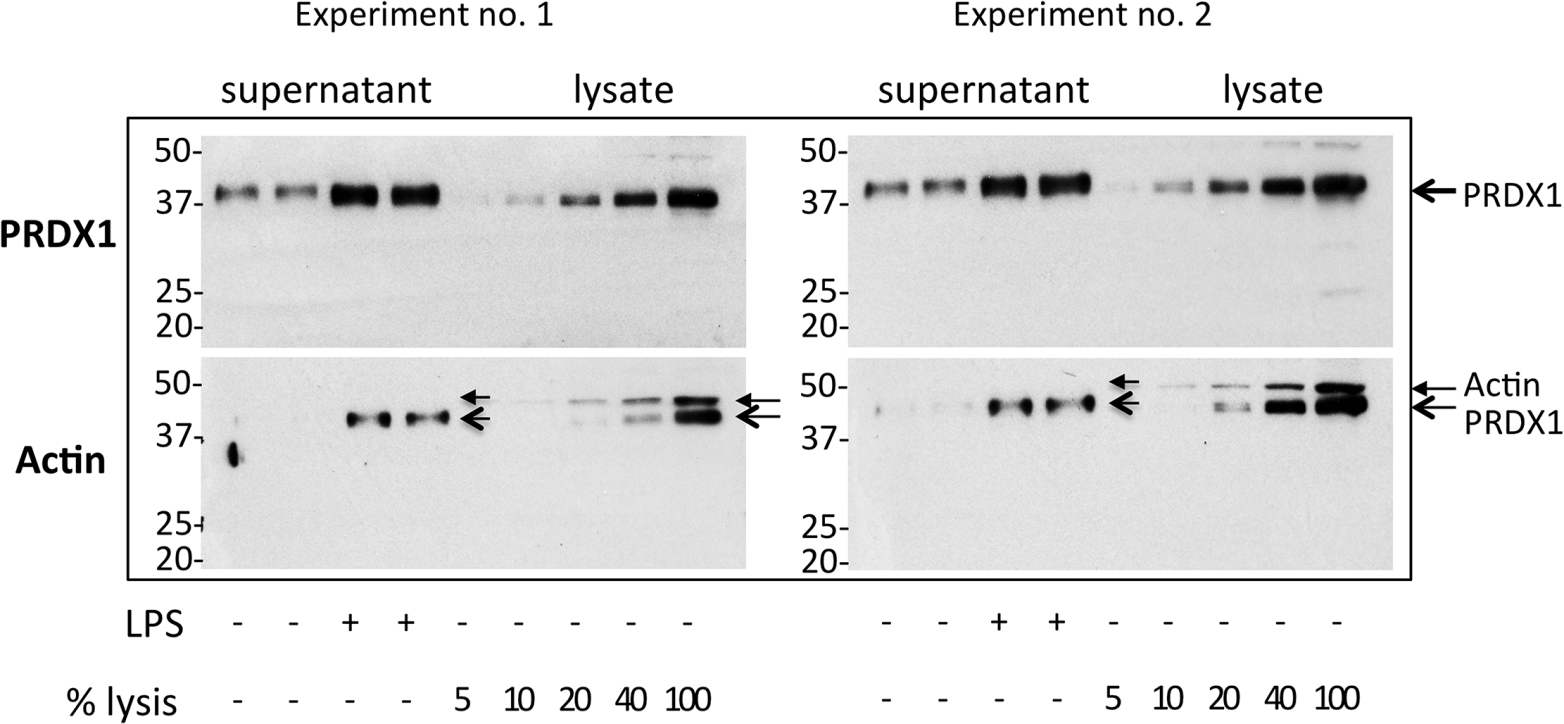
Cell lysis cannot account for LPS-induced release of PRDX1. Western blot analysis following non-reducing SDS-PAGE (12% acrylamide) of RAW264.7 supernatants obtained from cells cultured with or without 100 ng/ml LPS for 24 h (1 x10^6^ cells in 6 well plates in 1ml of OPTI-MEM) and of different percentages of cell lysate obtained from the same number of cells, cultured without LPS, resuspended in 1ml sample buffer (to mimic a supernatant containing the maximum amount of PRDX1 that would be released if 5, 10, 20, 40 or 100% of the cells were necrotic). Top, Western blot for PRDX1. Bottom, the same membrane was stripped and reprobed with anti-actin as a reference intracellular protein. Arrows indicate the position of Actin and PRDX1. Please note that some residual PRDX1 was still detected after stripping (bottom gels).

Finally, we designed an experiment to calculate the percentage of cell lysis that would be required to fully explain the quantity of proteins detected in the supernatant. For this purpose we treated cells in 1 ml of culture medium for 24 h with and without LPS and collected the supernatant. In parallel wells, the same number of cells was lysed in the same volume (1 ml) of SDS sample buffer. We then loaded the same volume (25 μl) of supernatant or of cell lysate at different dilutions corresponding to different percentages of the lysate (25 μl of undiluted lysate would represent a 100% cell lysis, a 1:2.5 dilution would correspond to a 40% cell lysis and so on). A Western blot for PRDX1 was then performed. As shown in Fig 2, more then 40% of cell lysis would be necessary to explain the amount of PRDX1 detected in LPS-stimulated supernatants. The membrane was then stripped and reprobed with anti-beta actin, as a loading control for cell lysate. Increasing percentages of cell lysates correlate with increasing amounts of actin, and this was not detectable in the supernatants.

Proteins identified in supernatants from BioGEE-preloaded, LPS-treated RAW264 macrophages purified on streptavidin-agarose. Full set of data is reported in S1 Table. Ticks indicate the proteins that are unique to the BioGEE samples as opposed to the untreated control (S1 Table), or (last column) those that were also identified in bands from electrophoretic separation and listed in S2 Table.

The full set of data, with the list of proteins identified in samples with and without BioGEE are available in the Supporting information.

### Confirmation of glutathionylation of secreted redoxins by Western blot analysis

The experimental design used should, in theory, specifically identify glutathionylated proteins. However, it is possible that some proteins might bind to streptavidin beads non-specifically, i.e. even in the absence of glutathionylation, and in fact some proteins were identified in the first experiment even in the absence of BioGEE. Thus, we tried to confirm the glutathionylation by a different approach. We have reported previously that PRDX2 released by LPS-stimulated RAW264.7 macrophages is glutathionylated [26]. In this study, we performed an immunoprecipitation for PRDX1 or TXN1, followed by Western blotting with streptavidin-peroxidase.

**Fig 3:**
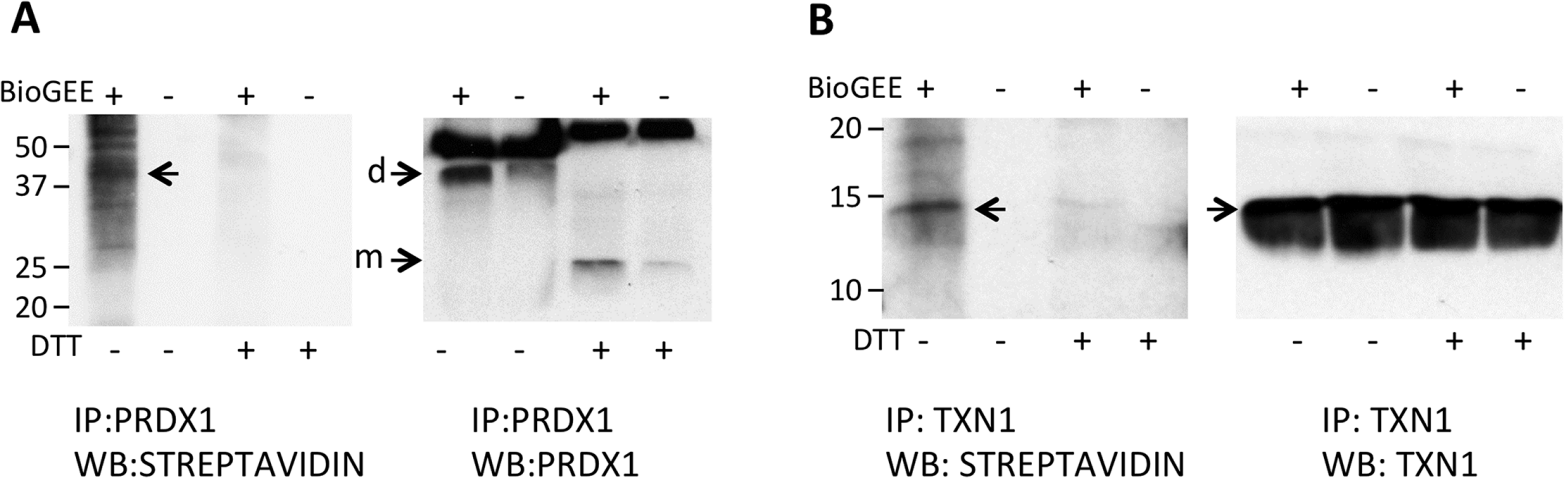
Proteins released in glutathionylated form. Proteins in the NEM-blocked supernatants from BioGEE-pretreated, LPS-stimulated cells were immunoprecipitated with anti-PRDX1 (A) or anti-TXN1 (B). Immunoprecipitated proteins were run under non-reducing (two lanes on the left) or reducing conditions (the two lanes with DTT, on the right). Proteins were then visualized by Western blot with streptavidin peroxidase. The same blot was stripped and reprobed with anti-PRDX1 or anti-TXN1 antibody to locate the proteins (left, in both A and B). m, monomer; d, dimer.

As shown in Fig 3A, anti-PRDX1-immunoprecipitated samples, when blotted with streptavidin-peroxidase identified a band compatible with the PRDX1 dimer. Stripping the membrane and re-probing with anti-PRDX1 confirmed the identity of this band as the PRDX1 dimer. When samples had been previously reduced with DTT, the band visualized with streptavidin disappeared, indicating the presence of a mixed disulphide with BioGEE. Of note, when the DTT-reduced sample was stained with anti-PRDX1 antibody, only the monomeric form of the protein was visible.

We could also confirm, using the same experimental approach, the glutathionylation of secreted TXN1 (Fig 3B). Streptavidin-peroxidase revealed only one band, of a molecular weight compatible with TXN1 that was not present in samples reduced with DTT. Immunostaining of the same membrane with anti-TXN1 antibody confirmed the identity of the band as TXN1.

### Release of redoxins from influenza virus-infected cells

We investigated whether influenza virus infection caused release of redoxins. For this purpose we infected A549 human lung epithelial cells with Influenza PR8 virus (4 multiplicity of infection units for 24 h, conditions in which we could detect virus production, 6 ± 2 hemagglutinin units), and analyzed supernatants and cell lysates by Western blot following electrophoresis under non-reducing conditions.

**Fig 4:**
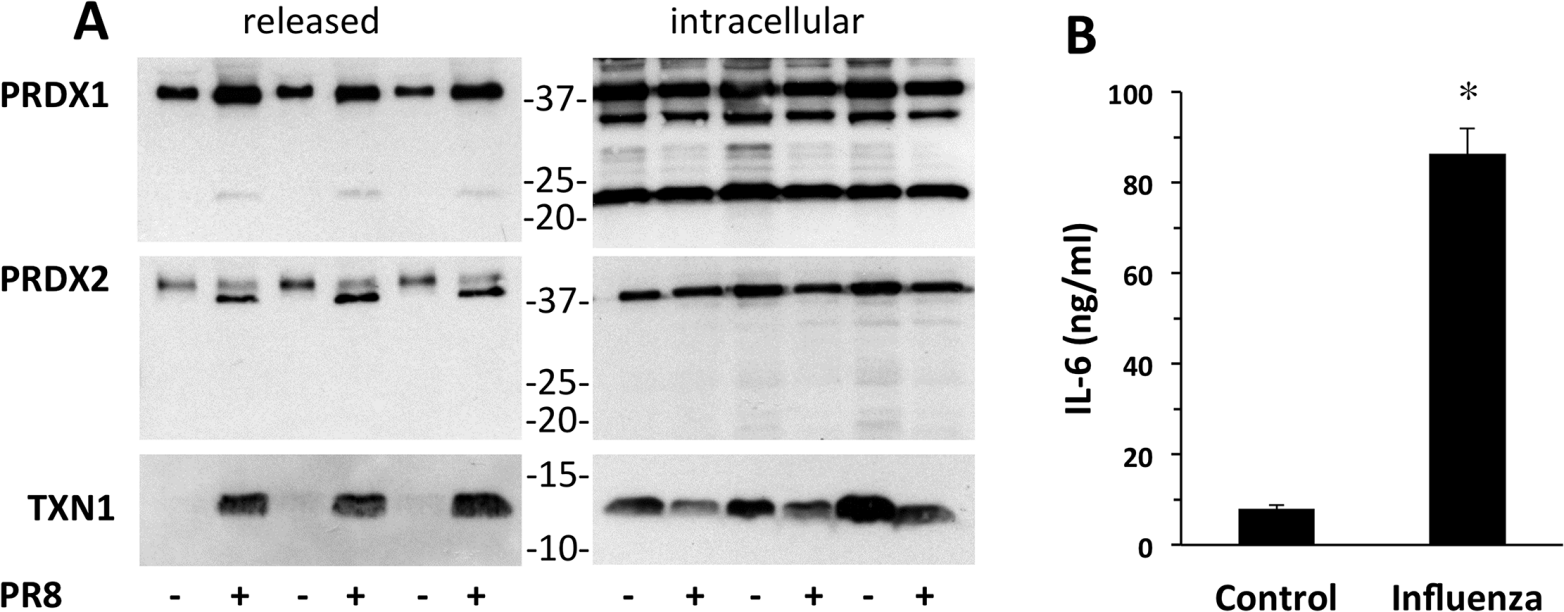
Influenza virus induces release of redoxins. Western blot analysis following non-reducing (12% for PRDXs and 15% for TXN1) SDS-PAGE of A549 supernatants and cell lysates. A549 cells were infected with PR8 virus (+) or mock-infected (-) for 24 h as described in the Methods. Cell lysates were also analyzed after blocking with 40 mM NEM. B. IL-6 levels as measured by ELISA. Data are mean ± SE from three independent samples. *P<0.01 vs. non-infected cells by Student’s t-test.

As shown in Fig 4A, infected cells released higher levels of PRDX1 and PRDX2 compared to control cells. In these experiments, control cells had a considerable basal release of PRDX1 and PRDX2 when compared with RAW264.7 macrophages, although this may be due to the different cell type. Also in this case, PRDX1 and PRDX2 were released only in the dimeric form. Interestingly, the PRDX2 dimer runs as a doublet in influenza-infected cells, but as a single band in non-infected controls.

Release of TXN1 was also markedly induced by viral infection while, unlike PRDX1 and PRDX2, little or no TXN1 was released in non-infected cells. This was associated with a visible decrease in the intracellular TXN1 levels. Influenza-infected cells also showed a marked increase in IL-6 production (Fig 4B) as reported previously [36].

**Fig 5:**
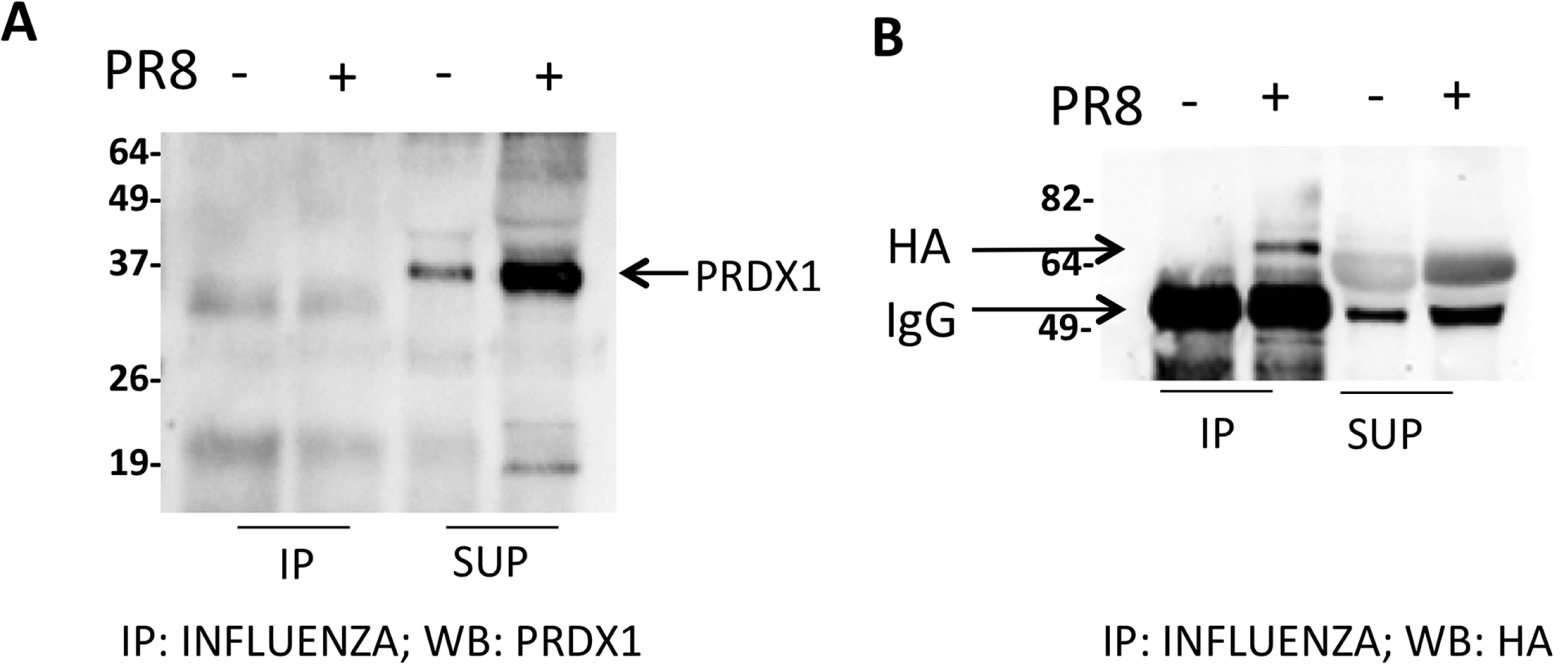
PRDX1 is not released associated with influenza virions. A549 cells were infected (+) or mock infected (-) as described in the Methods. Supernatants were blocked with 40 mM NEM, concentrated and the virions immunoprecipitated with anti-influenza antibody. Immunoprecipitated samples and supernatants left after immunoprecipitation were run under non-reducing (A) or reducing conditions (B) and analyzed by Western blot with anti-PRDX1 (A) or anti-HA (B) antibody.

We also wondered whether redoxins could be secreted by infected cells present in the viral particles. For this purpose, we immunoprecipitated the viral particles with an anti-influenza antibody and analyzed the supernatant and the immunoprecipitate by Western blot. As shown in Fig 5, PRDX1 was only detected in the supernatant but not in the immunoprecipitate. As a control, we also measured the influenza hemagglutinin (HA) that, as expected, was present in the immunoprecipitate from infected cells (but not in mock infected) to indicate that immunoprecipitation was effective. From these data one can conclude that PRDX1 is not released associated with the virions.

### Inhibition of LPS-induced release of redoxins and PFN1 by dexamethasone and thiol antioxidants

**Fig 6:**
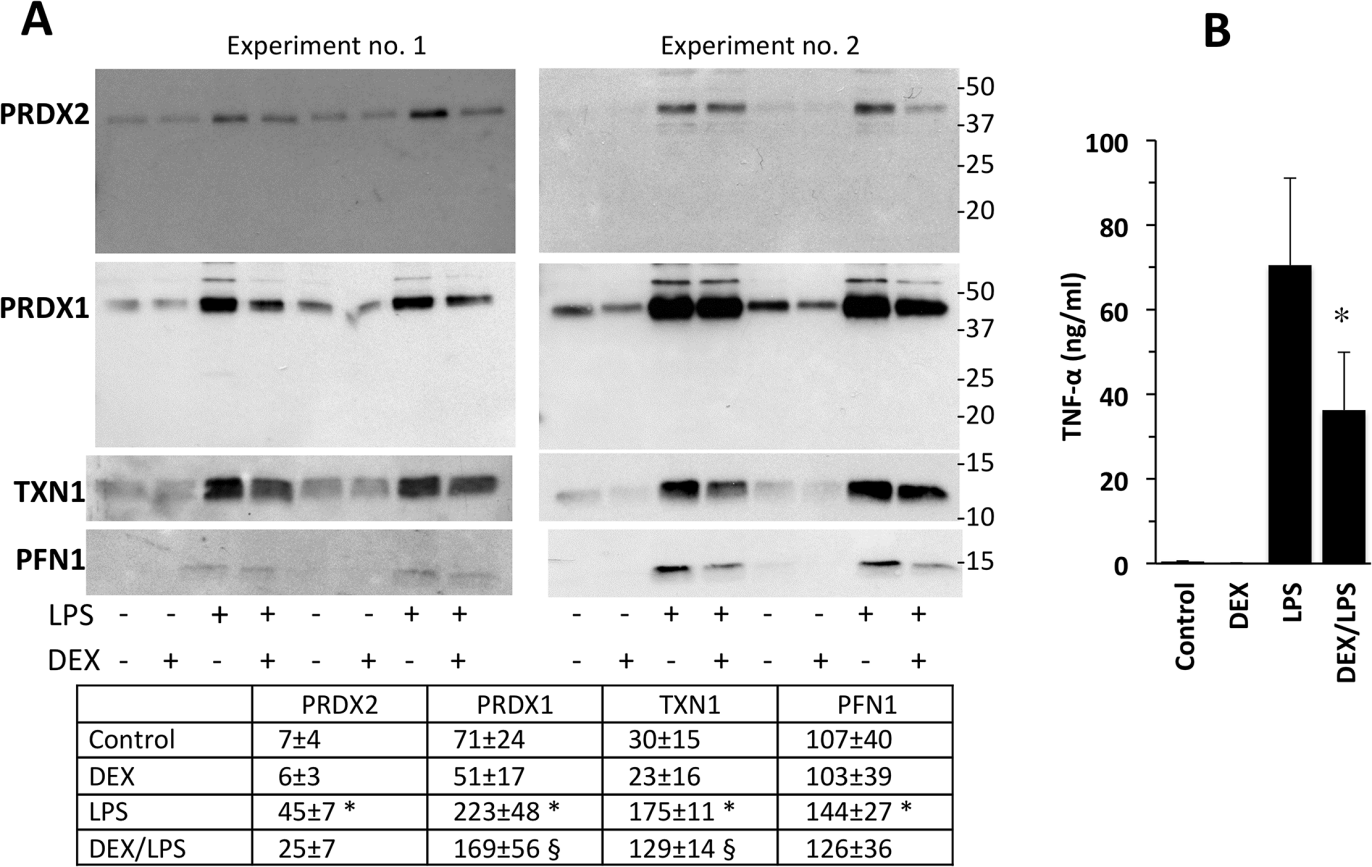
LPS-induced protein release is down-regulated by DEX. Experiments were performed as described in the legend to Fig 2 except that, when indicated, 10 μM DEX was present during the 24-h culture. A. Supernatants were run under non-reducing conditions after blockade with NEM. Data for the densitometric analysis are expressed as arbitrary units and are the mean ±SE (n = 4). * P<0.05 vs. control, § P<0.05 vs. LPS alone by Student’s t-test for paired data. B. TNF-α levels as measured by ELISA. Data are mean ± SE from three independent samples. *, P<0.01 vs. LPS alone by Student’s t-test.

We investigated the effect of DEX and thiol antioxidants (GSH-C4 and NAC) on the secretion of PRDX2 and other redoxins. The effect of 10 μM DEX on the release of these proteins is shown in Fig 6A. It can be seen that in both experiments DEX inhibited, although to a different extent, the release of most of the proteins studied. We also measured TNF-α levels, as a reference LPS-induced cytokine, in the same supernatants. The results of the ELISA shown in Fig 6B indicate that, under these experimental conditions, DEX lowered TNF-α production by 50%.

**Fig 7:**
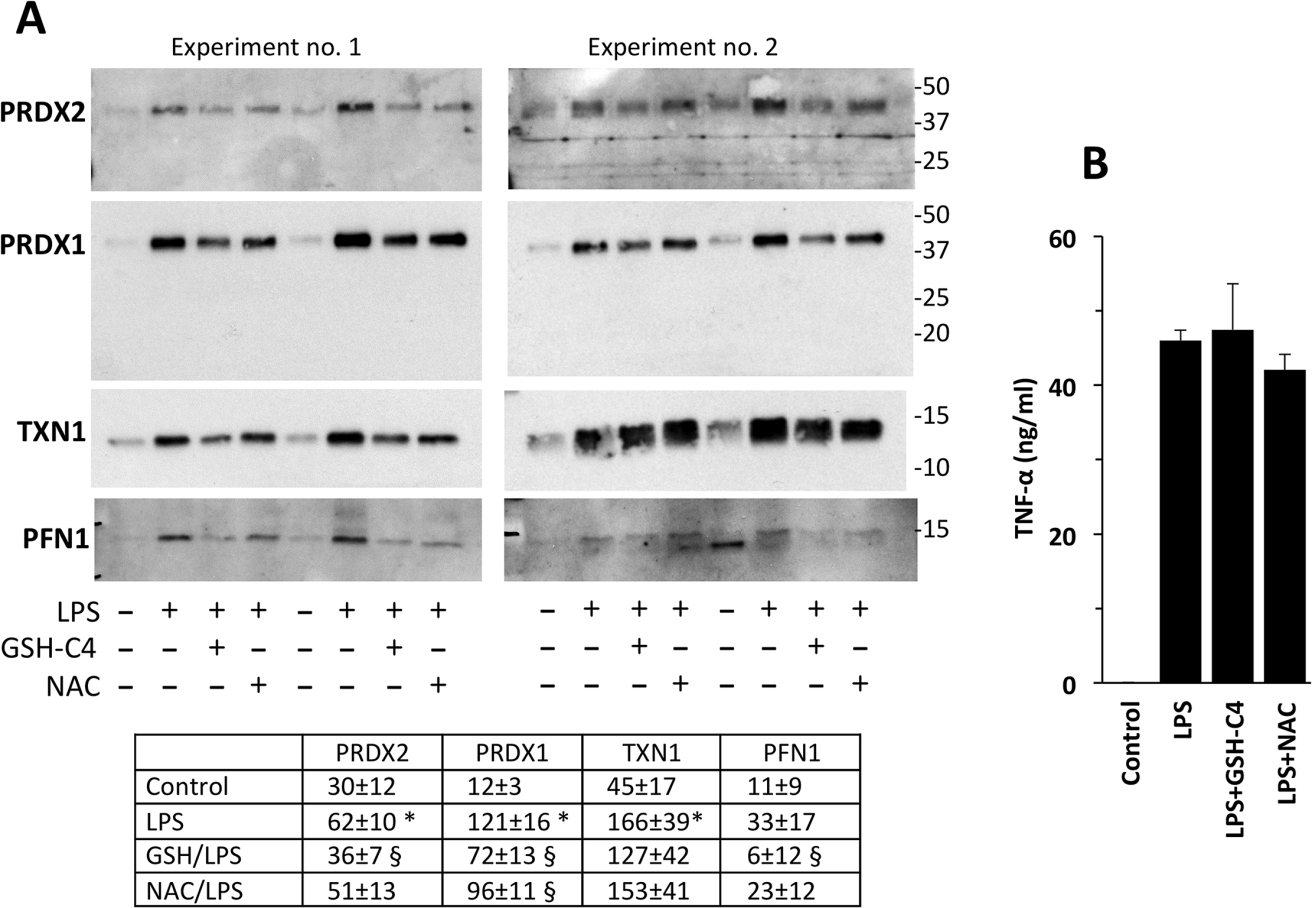
LPS-induced protein release is down-regulated by thiol compounds. Cells were pre-treated with 10 mM GSH-C4 or NAC for 2 h, washed and then LPS was added for 24 h. A. Supernatants were run under non-reducing conditions after blockade with NEM. Data for the densitometric analysis are expressed as arbitrary units and are the mean ± SE (n = 4). * P<0.05 vs. control, § P<0.05 vs. LPS alone by Student’s t-test for paired data. B. TNF-α levels as measured by ELISA. Data are mean ± SE from three independent samples.

We then tested NAC, added at the concentration of 10 mM, together with 1–100 ng/ml LPS. PRDX2 was analysed by Western blot but the results of this preliminary experiment were difficult to interpret. In fact, while LPS induced a dose-dependent release of PRDX2 dimer, only the monomer was detected when NAC was present (S5 Fig). It is likely that NAC acts as a reducing agent, reducing the PRDX2 dimer after its release. We thus studied the effect of GSH-C4 and NAC using a 2-h pretreatment, to allow entry into cells, then washing the cells to eliminate residual thiols from the supernatants, before the addition of LPS for 24 h. As shown in Fig 7A, GSH-C4 significantly inhibited the release of PRDX1 and PRDX2, while NAC was less effective. Under these experimental conditions, production of TNF-α was not affected (Fig 7B).

## DISCUSSION

Using redox proteomics to identify glutathionylated proteins in the secretome of LPS-stimulate macrophages, we identified a number of proteins, some of which have not been previously described, released in response to LPS or to influenza virus infection. Our proteomics identification was validated using specific antibodies to these proteins by Western blot, demonstrating that they are in fact released by macrophages in response to LPS, or by epithelial cells in response to influenza virus infection.

Previous studies on identification of glutathionylated proteins have focused on the analysis of soluble proteins located in the cell lysates, predominantly cytoplasmic proteins [37, 38]. In fact, it is often thought that cytosolic proteins are rich in free thiol groups and have few structural disulfides because of the highly reducing environment in the cytoplasm, while extracellular proteins have fewer free thiols and are rich in disulfide bonds because the extracellular environment is an oxidizing one [39, 40]. Free thiols of cytosolic protein can form reversible disulfides [23, 41, 42], including those with GSH. Secreted proteins with a reactive cysteine, such as serum albumin, can be found in the serum in the cysteinylated form, rather than glutathionylated [43], because extracellularly the cysteine/cystine couple is present at higher concentrations than that of GSH/GSSG, the opposite situation to that observed in the cytoplasm. It was therefore surprising to find secreted glutathionylated proteins, and it may be that the glutathionylation takes place in the cytoplasm before the release of the protein. On the other hand, it should be noted that at least one plasma protein, transthyretin, although it is predominantly present in cysteinylated form, could also be found, although to a lesser extent, as a glutathionylated species [44].

Protein databases usually identify proteins as “secreted” based on the presence of a signal sequence for targeting to the Golgi [45]. Thus, proteins released via non-classical secretion are not generally tagged as secreted proteins in the databases. In fact, none of the proteins investigated in this paper (PRDX1, PRDX2, PFN1, TXN1, VIM) have a signal peptide, according to the database UNIPROT (http://www.uniprot.org/). Interestingly, these proteins had all been previously reported to be either present in the secretome or in circulation. We previously reported that PRDX2 is secreted by macrophages in response to LPS [26]. Secretion of PRDX1 by various cell lines has also been reported [46], and both PRDX1 and 2 are increased in the serum of diabetic patients with peripheral atherosclerotic disease [47] or in the secretome of cancer cells [48]. Release of TXN1 by activated macrophages has also been described [49] and TXN1 levels are elevated in HIV patients [50]. A previous report had also shown that VIM is secreted by activated macrophages [51].

We were able to formally demonstrate the glutathionylation of PRDX1 and TXN1 by immunoprecipitation, in addition to that already reported for PRDX2 [26]. We were however unable to obtain such evidence for secreted PFN1 and VIM, due to the low quality of the Western blot obtained with the antibodies available. This may be due to the fact that low levels of secreted proteins are present in the macrophage secretome. It should be noted, however, that these proteins were previously reported to be susceptible to glutathionylation, even if those results were obtained with intracellular, not secreted, proteins [23].

Another important observation was that PRDX1 is secreted mainly as a disulfide-linked dimer, as we previously observed for PRDX2 [26], while we have no evidence of dimer formation with any of the other proteins investigated. Of note, despite in vitro studies indicating that TXN1 can form disulfide-linked homodimers over several days [52], this has never been demonstrated to occur *in vivo*, and our data confirm that this does not happen, at least in our cell culture system.

A note of caution is advised in interpretation of the Western blot data obtained using anti-VIM antibodies. The data confirm the presence of the protein intracellularly and its secretion in response to LPS, but the apparent molecular weight does not correspond to the accepted value for VIM. Although we previously found VIM among the proteins undergoing glutathionylation [23] and others have reported that VIM is susceptible to cleavage by caspases [53], we believe that further experiments are needed to confirm its presence in the supernatant in our experimental conditions.

Dexamethasone, a classical anti-inflammatory agent which inhibits LPS-induced release of TNF in our experimental model, also inhibits the LPS-induced release of PRDX1, PRDX2, TXN1 and PFN1 (Fig 6) indicating that their release parallels that of other inflammatory mediators. The pattern of regulation was different with antioxidant thiols, GSH-C4 and NAC. As shown in Fig 7, these molecules, particularly GSH-C4, inhibited the LPS-induced release of PRDX1, PRDX2, PFN1 and, to a lesser extent, TXN1, but did not affect that of TNF-α. This finding supports the idea that the release of these proteins is more susceptible to redox regulation than that of TNF-α.

Another major difference with respect to inflammatory cytokines, such as TNF-α, is that all the proteins identified are normally present in the cell and their expression is not induced by LPS. This is very similar to the behaviour of HMGB1, a danger signal that is released in response to LPS from a preformed pool [4]. Obviously, all the proteins we identified will also be released as a consequence of cell death by necrosis, along with any other cytoplasmic proteins. Release of proteins by necrosis and subsequent recognition of these proteins as danger signals that induce an inflammatory response has been described for HMGB1 [54], ATP [55], and various PRDXs [56].

However, the release of these proteins reported here is not simply secondary to cell necrosis. First of all, LPS-treated cells are not dead and actively respond by inducing TNF-α production, as shown here. Furthermore, cell viability as assessed by CellTiter-Blue, an assay that would be able to detect a 5% toxicity, was not significantly affected by LPS (viability 99.6%). These findings were confirmed using a different assay of cell viability (CellTitre Glo). More convincingly, by loading different amounts of a cell lysate we could extrapolate that, based on PRDX Western blots, only the lysis of more than 40% of the cells would account for the levels of PRDX1 secreted.

These findings open the question of the biological relevance of the secretion of these proteins. Of the proteins investigated, we focused our attention on redoxins (PRDX1, PRDX2, TXN1) because they are already known to have inflammatory or cytokine-like activities. In particular, PRDX1 and PRDX2 can stimulate production of inflammatory cytokines [26, 56, 57] and activate NK cells [58]. TXN1 has also cytokine-like properties, including upregulation of IL-2 receptor [59] and chemotactic activity [60]. Furthermore, as PRDXs are thioredoxin peroxidases, these proteins belong to the same metabolic pathway, and we speculate that they could carry out peroxidatic reactions and thiol-disulfide exchange in the extracellular milieu, particularly in inflammatory conditions when hydrogen peroxide may also be present [26].

We also report that influenza virus-infected epithelial cells release redoxins. It is known that viruses, including influenza, can induce oxidative stress and this can be important in activating intracellular signalling pathways involved in viral replication as well as cytokine production [61–64]. An exaggerated inflammatory response to some highly virulent strains, due to mechanisms yet to be defined, is thought to be responsible for higher mortality associated with these infections [65], and it may well be that factors such as oxidative stress could play an important pathogenic role. It is possible that redoxins are associated with the viral particles. Glutaredoxin has been detected within HIV-1, even if its very low levels led the authors to speculate a passive incorporation by the virus [66]. Our data on immunoprecipitated viral particles suggest that this is not the case, at least for PRDX1, although we cannot definitely rule out that a small amount of the protein could be passively incorporated into virions, or that this could happen for other proteins.

Further studies will be required to determine the possible mechanisms by which these proteins are secreted. As discussed above, none of these proteins have signal peptides and so must be secreted via a non-classical route. We recently showed that the secretion of two of the proteins studied here, PRDX1 and 2, was dependent on oxidation of cysteine residues to form disulphide-linked homodimers [35]. Although none of the other proteins described here (TXN1, PFN1 and VIM) were detected as dimers in our experimental conditions, it is still possible that other forms of cysteine oxidation (for example glutathionylation) may be implicated in their release.

In conclusion, using redox proteomics we have identified a number of proteins released in response to LPS and influenza virus. Further studies will be required to investigate the possible role of the proteins identified here, as well as to validate all the 46 proteins identified by redox proteomics analysis. While some may have immunoregulatory effects, in a manner similar to that of the PRDXs and TXN1, other proteins may be useful as biomarkers of oxidative stress associated with inflammation. Identifying proteins secreted under specific oxidative conditions may in fact provide more information than that obtained by measuring the absolute concentration of a protein, as it has recently been shown for pentraxin 3, that can also be oxidized to form disulfide-linked oligomers that represent a better biomarker for sepsis [67]. Development of new techniques to detect specific oxidation forms of the proteins described here is currently under way and these assays may provide novel biomarkers of diseases associated with oxidative stress.

## MATERIALS AND METHODS

### Reagents

Lipopolysaccharide (LPS) from *E*. *coli* 055:B5 was from Sigma. Influenza A/Puerto Rico/8/34 H1N1 virus (PR8) was grown in the allantoic cavities of 10-day-old embryonated chicken eggs and harvested after 48h at 37°C. Glutathione ethyl ester, biotin amide (BioGEE, Invitrogen) was dissolved in DMSO and then diluted to a final concentration of 200 μM in cell-culture medium. Dexamethasone 21-phosphate disodium salt, N-acetyl-L-cysteine, and N-ethylmaleimide were from Sigma-Aldrich. GSH-C4 was from Pepnome Limited, Hong Kong, China.

### Cell cultures

RAW264.7 mouse macrophages were cultured in RPMI 1640 medium (Sigma Aldrich) with 10% FBS (Invitrogen). Treatment with BioGEE and/or LPS was performed in serum-free OPTI-MEM I medium (Invitrogen). Cells were plated in 6-well plates (1 x 10^6^ in 3 ml of RPMI with 10% FBS). One day later, medium was changed to 1 ml of OPTI-MEM. Where indicated, BioGEE was added to a final concentration of 200 μM 1 h before LPS stimulation (100 ng/ml, for 24h). DEX was used at 10 μM for 24h. NAC and GSH-C4 were used at 10 mM for 2h and removed by washing with PBS before performing LPS stimulation as describe above. Supernatants were collected and N-ethylmaleimide (NEM) added to a final concentration of 40 mM (from a 1M stock solution) to avoid thiol-disulfide exchange. Cells were lysed with sample buffer containing 40 mM NEM. Cell viability was measured in 96-wells microtiter plates using CellTiter-Blue assay (Promega). A549 human lung epithelial cells were cultured in DMEM medium (Sigma Aldrich) with 10% FBS (Invitrogen) and infected with PR8 at a multiplicity of infection (MOI) of 4, incubating the cells with the virus for 1h at 37°C in serum-free medium, washing with PBS and then adding medium with 2% FBS for 24h. Virus production was determined in cell supernatants by measuring hemagglutinin units as described previously [31].

### Cell viability assays

Cells were plated at 25,000/well in 96-well plates in RPMI + 10% FBS. After overnight culture, the medium was replaced with serum-free OPTI-MEM I and the cells were incubated with or without 100 ng/ml of LPS. After 24 h, cell viability was measured with CellTiter-Blue or CellTiter-Glo (both from Promega), following the instructions of the manufacturer.

### Mass spectrometry and data analysis

RAW264.7 cells were cultured in 6-well plates in 1.5 ml of OPTI-MEM as described above. Two wells were pre-loaded with BioGEE and then treated with LPS as described above, the other two wells were treated identically except that they were not pretreated with BioGEE. Twenty-four hours after addition of LPS, the supernatants from the two wells were collected and combined, NEM added to a final concentration of 40 mM, and concentrated to 300 μL using 5 kDa-cutoff Vivaspin columns (GE Healthcare) at 4°C. The concentrated proteins were then mixed with 50 μL of streptavidin-agarose (Sigma) and incubated under rotation for 30 min at 4°C. The beads were then washed with cold PBS, followed by two washes with cold PBS containing 0.1% SDS, and then with 25 mM NH_4_CO_3_. Proteins attached to the beads were then subjected to 2,2,2-Trifluoroethanol-enhanced trypsin digestion *in situ*, essentially as described [68]. Following addition of trypsin, samples were incubated for 18 hours at 37°C, and the digestion reaction was quenched by the addition of TFA to 0.1% prior to LC MS/MS analysis as described below.

For the SDS-PAGE fractionated samples (second experiment), the resulting gel bands were excised and subjected to trypsin in-gel digestion essentially as previously described [69]. The supernatant from the digested samples was removed and acidified to 0.1% TFA, dried down, and reconstituted in 0.1% TFA prior to LC-MS/MS analysis.

In both cases, for LC-MS/MS the resulting peptides were fractionated on a 250 mm x 0.075 mm reverse phase column using an Ultimate U3000 nano-LC system and a 2 hour linear gradient from 95% solvent A (0.1% formic acid in water) and 5% B (0.1% formic acid in 95% acetonitrile) to 50% B at a flow rate of 250 nL/min. Eluting peptides were directly analysed by tandem MS using a LTQ Orbitrap XL hybrid FTMS (ThermoScientific) and the derived MS/MS data searched against the ipi. MOUSE.v3.72 database (56957 entries) [70] using Sequest version SRF v.5 as implemented in Bioworks v3.3.1, assuming carboxyamidomethylation (Cys), deamidation (Asn) and oxidation (Met) as variable modifications and using a peptide tolerance of 10ppm and a fragment ion tolerance of 1.0 Da. Filtering criteria used for positive protein identifications are Xcorr values greater than 1.9 for +1 spectra, 2.2 for +2 spectra and 3.75 for +3 spectra, a delta correlation (DCn) cut-off of 0.1 and at least two unique peptides.

### Western blot analysis and immunoprecipitation

Supernatants, after blocking thiols by alkylation with NEM, were centrifuged to eliminate cellular debris and analyzed by SDS-PAGE under non-reducing or reducing conditions (by addition of 10 mM DTT), followed by Western blotting. According to the molecular weight of the protein of interest, gels with different percentage of acrylamide were used (ranging from 7.5% to 15%). Biotinylated proteins were visualized using streptavidin peroxidase (Roche) at 1:25,000 dilution. Western blot for other proteins used the following antibodies,: anti-PRDX1 (Abcam, cat. 41906), anti-PRDX2 (prepared against human PRDX2 but cross-reacting with mouse PRDX2, as described elsewhere [71]) at 1:1000 dilution, anti-mouseTXN1 (IMCO) at 1:2000 dilution, Anti-VIM (Sigma, cat. AV48225), at 1:500 dilution, anti-PFN1 (Sigma, cat P7624) at 1:500 dilution. Membranes were then incubated with an anti-rabbit HRP-linked secondary antibody (Sigma, cat. A0545) at 1:25,000 dilution and developed using ECL WB Analysis System (GE Healthcare). For immunoprecipitation experiments, supernatants from BioGEE labelled, LPS-stimulated RAW264.7 cells, blocked with NEM, were concentrated using 5 kDa Vivaspin columns and incubated with anti-PRDX1 (Abcam, cat. 41906) or anti-TXN1 (IMCO) at 1:100 dilution at 4°C for 4h; then the immunocomplex was precipitated with protein G agarose (Pierce) at 4°C for 18h. Agarose beads were washed three times with cold PBS and then eluted by boiling in sample buffer for 4 minutes. Eluate was split into two aliquots, one reduced with 10 mM DTT, one left untreated and both loaded on SDS-PAGE gels (12% and 15% acrylamide for PRDX1 and TXN1, respectively) followed by Western blot with streptavidin peroxidase as described above. The same blots were then stripped with stripping buffer (Thermo scientific) and reprobed with anti-PRDX1 and anti-TXN1. When indicated, densitometric analysis was performed on the Western blots, following scanning, using GeneTools (Syngene) version 4.02.01.

To immunoprecipitate influenza viral particles, supernatants from PR8-infected A549 cells were blocked with NEM, concentrated using 10 kDA vivaspin columns and incubated with anti-influenza antibody (MerckMillipore, cat. AB1074) at 1:100 dilution at 4°C for 4h; then the particles were immunoprecipitated with Protein A/G Plus-Agarose (Santa Cruz Biotechnology) at 4°C overnight. Agarose beads were washed four times with PBS and then eluted with sample buffer at 100°C for 4 minutes. Eluate was split into two aliquots, one run on SDS-PAGE under non reducing conditions and analysed in Western blot with anti-PRDX1 antibody, as described above; the other one was reduced with DTT, before electrophoresis and Western blot analysis with anti-HA antibody (Santa Cruz Biotechnology, cat. sc-52025) at 1:1000 dilution.

### ELISA for TNF-α and IL-6

Mouse TNF-α and human IL-6 were assayed by ELISA using a DuoSet kit from R&D Systems according to the manufacturer’s instructions.

## Supporting Information

S1 Fig Glutathionylated proteins released by RAW macrophages after stimulation with LPS. A. Cells were preloaded with BioGEE where indicated, then stimulated with 100 ng/ml LPS for 24h. The supernatants were reduced with 10 mM DTT for 15 min or left untreated, then loaded on a 12% SDS-PAGE followed by Western blot with streptavidin-peroxidase. B. RAW cells were incubated with Bio-GEE for 1 hour, the medium was then removed and cells were washed with OPTI-MEM and replaced with fresh medium containing 100 ng/mL of LPS. Cell supernatants were collected and NEM added to a final concentration of 50 mM. Supernatants were applied to a SDS-PAGE gel and probed with streptavidin peroxidase.
(TIF)

S2 Fig Gel electrophoresis of the eluates from streptavidin beads. The supernatant from BioGEE-preloaded, LPS-stimulated RAW264.7 cells was incubated with streptavidin agarose and then eluted with 10 mM DTT for 30 min at room temperature and then again with 10 mM DTT for 10 min at 100°C. The eluate was run on SDS-PAGE under reducing condition and stained with Coomassie Blue. The three bands indicated by numbers were cut and used for protein identification.
(TIF)

S3 Fig Stability of PRDX2 monomer in cell culture. Human recombinant PRDX2 was expressed in *E*. *coli* with an N-terminal His-tag and purified using nickel agarose beads. Purified PRDX2 was incubated overnight at 37°C with 5% CO_2_ in complete DMEM in the presence of adherent HEK 293T cells (lanes 1–3) or in complete DMEM in the absence of cells (lanes 4–6). Proteins were then loaded on a 12% acrylamide gel for Western blotting with anti-His antibody.
(TIF)

S4 Fig Sensitivity of CellTiter Blue for detection of cell viability. Cells were plated in 96 well plates at 25,000/well (100%) or at the indicated percentage, down to 15,000 cells/well (60%). After overnight incubation, cells were treated with LPS 100 ng/ml (LPS) or medium alone (ctr) and incubated for 24 hrs. Then CTB (20 μl /well) was added and fluorescence detected after 3h. Results are the mean ± SD of quadruplicate samples. * P < 0.05 vs ctr; ***P < 0.001 vs ctr by Student’s t-test.
(TIF)

S5 Fig Effect of 10 mM NAC on PRDX2 release by LPS-stimulated RAW264.7 cells. Experiment was carried out as in the legend to Fig 2, except that different concentrations of LPS were used (0–100 ng/ml), with or without 10 mM NAC present during the entire 24-h culture.
(TIF)

S1 Table Proteins identified in the 3 bands excised from the gel shown in S1 Fig. (XLSX)

S2 Table Proteins identified in the samples prepared without or with BioGEE, following affinity purification on streptavidin beads. (XLSX)
