# Supplementary figures and images for: Redox Proteomics of the Inflammatory Secretome Identifies a Common Set of Redoxins and Other Glutathionylated Proteins Released in Inflammation, Influenza Virus Infection and Oxidative Stress
Source: PLoS One. 2015 May 18;10(5):e0127086. doi: 10.1371/journal.pone.0127086 (PMC4436175; doi:10.1371/journal.pone.0127086)

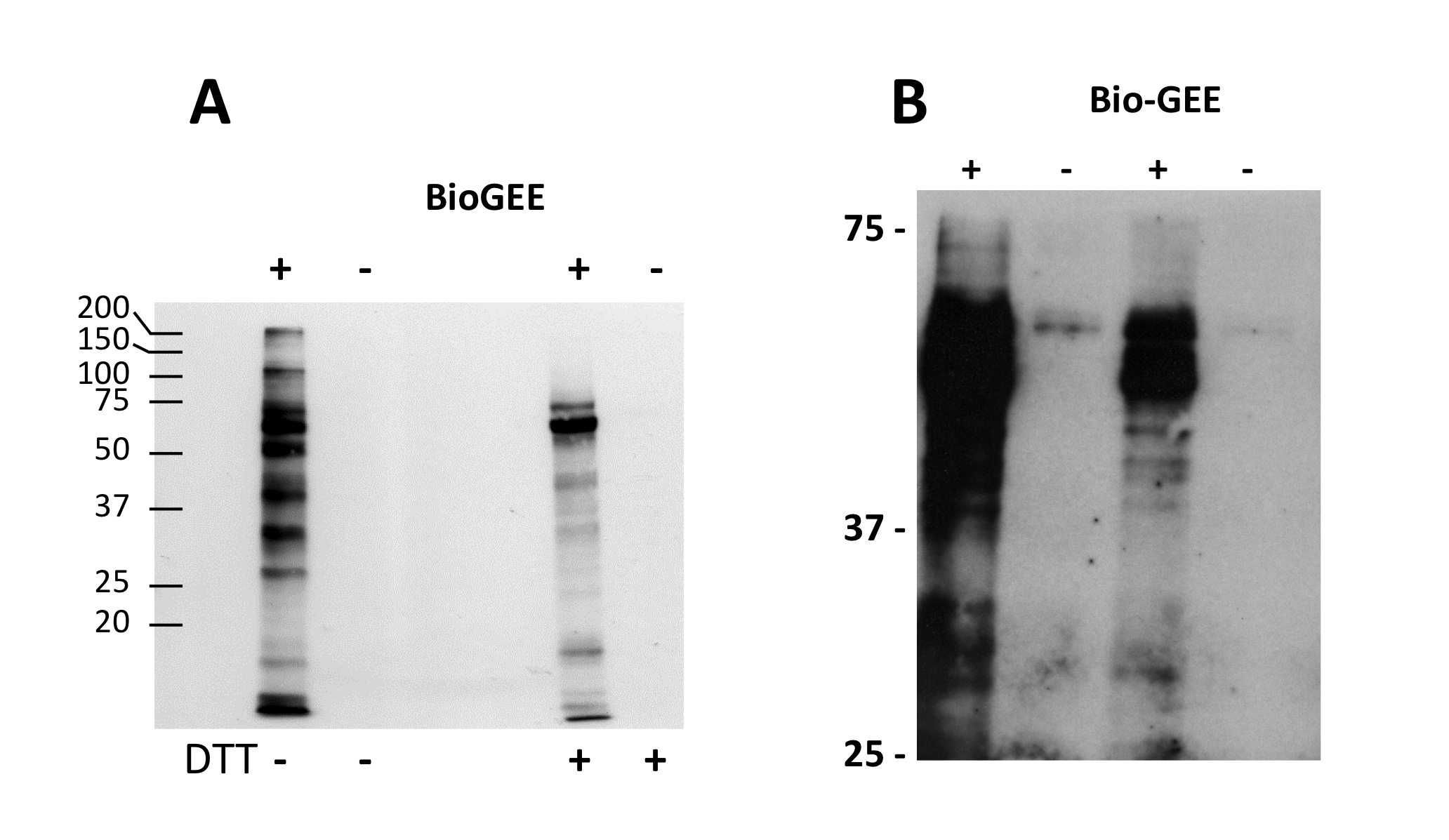

Supplement: S1 Fig — A. Cells were preloaded with BioGEE where indicated, then stimulated with 100 ng/ml LPS for 24h. The supernatants were reduced with 10 mM DTT for 15 min or left untreated, then loaded on a 12% SDS-PAGE followed by Western blot with streptavidin-peroxidase. B. RAW cells were incubated with Bio-GEE for 1 hour, the medium was then removed and cells were washed with OPTI-MEM and replaced with fresh medium containing 100 ng/mL of LPS. Cell supernatants were collected and NEM added to a final concentration of 50 mM. Supernatants were applied to a SDS-PAGE gel and probed with streptavidin peroxidase. (TIF) [file pone.0127086.s001.tif]

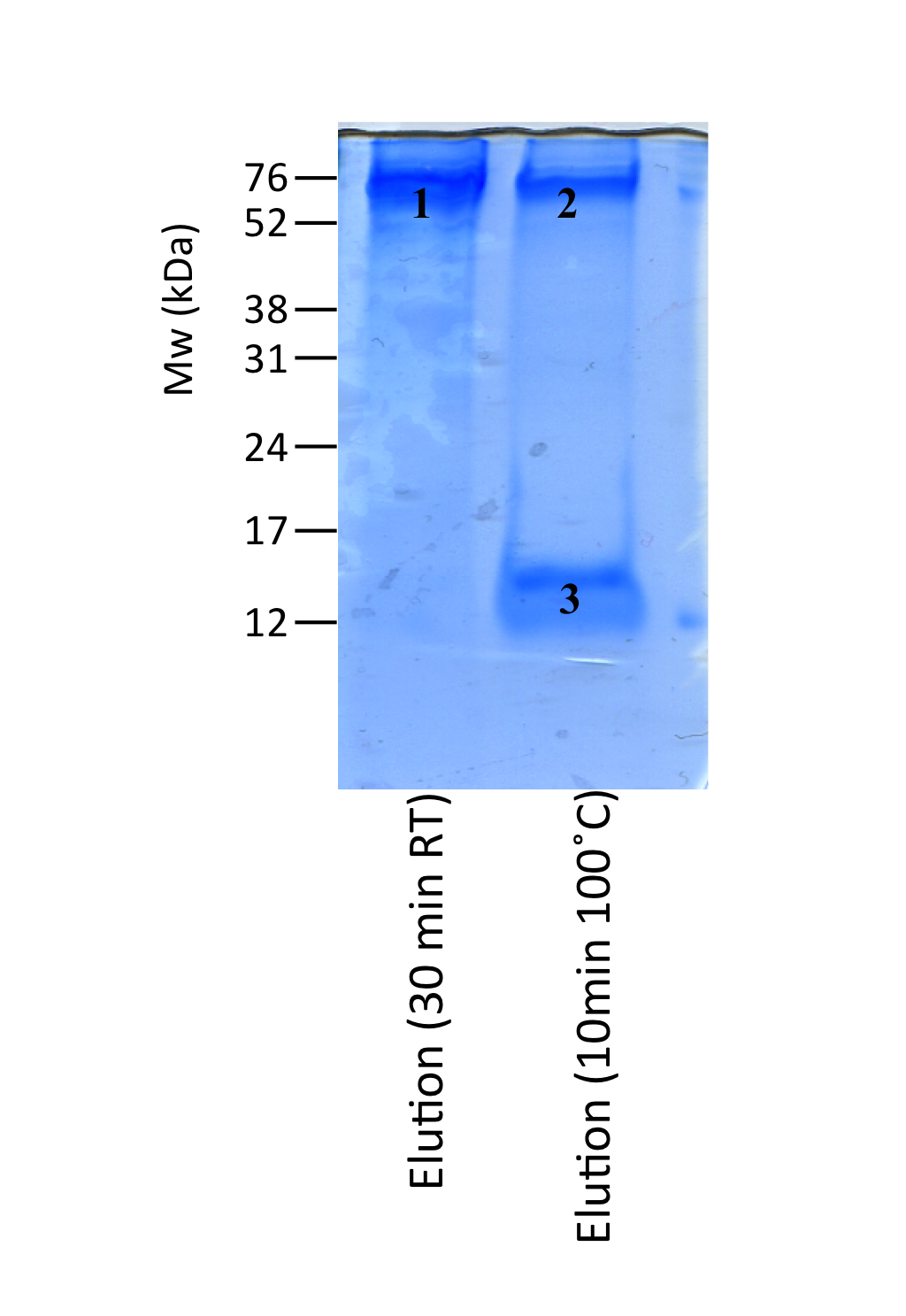

Supplement: S2 Fig — The supernatant from BioGEE-preloaded, LPS-stimulated RAW264.7 cells was incubated with streptavidin agarose and then eluted with 10 mM DTT for 30 min at room temperature and then again with 10 mM DTT for 10 min at 100°C. The eluate was run on SDS-PAGE under reducing condition and stained with Coomassie Blue. The three bands indicated by numbers were cut and used for protein identification. (TIF) [file pone.0127086.s002.tif]

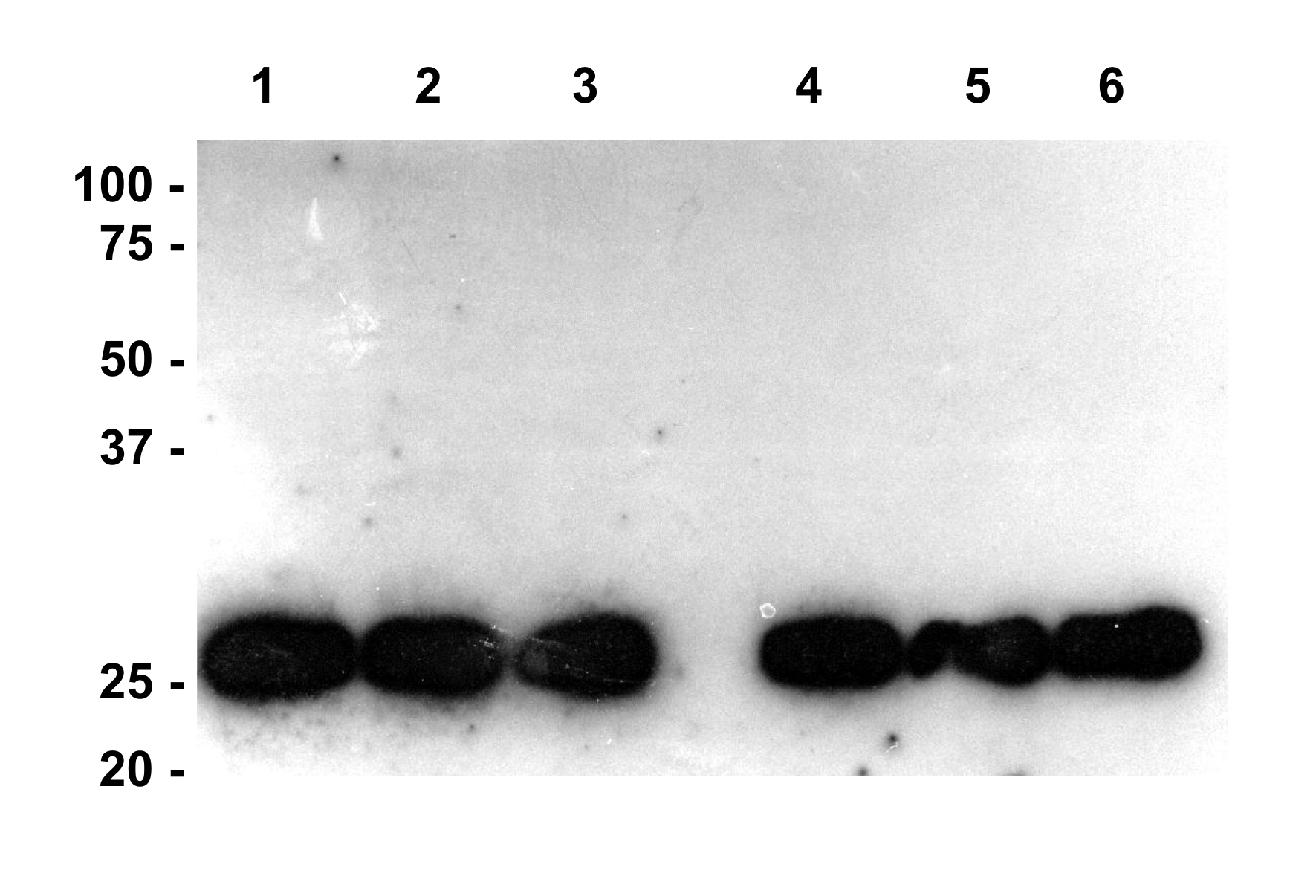

Supplement: S3 Fig — Human recombinant PRDX2 was expressed in E. coli with an N-terminal His-tag and purified using nickel agarose beads. Purified PRDX2 was incubated overnight at 37°C with 5% CO2 in complete DMEM in the presence of adherent HEK 293T cells (lanes 1–3) or in complete DMEM in the absence of cells (lanes 4–6). Proteins were then loaded on a 12% acrylamide gel for Western blotting with anti-His antibody. (TIF) [file pone.0127086.s003.tif]

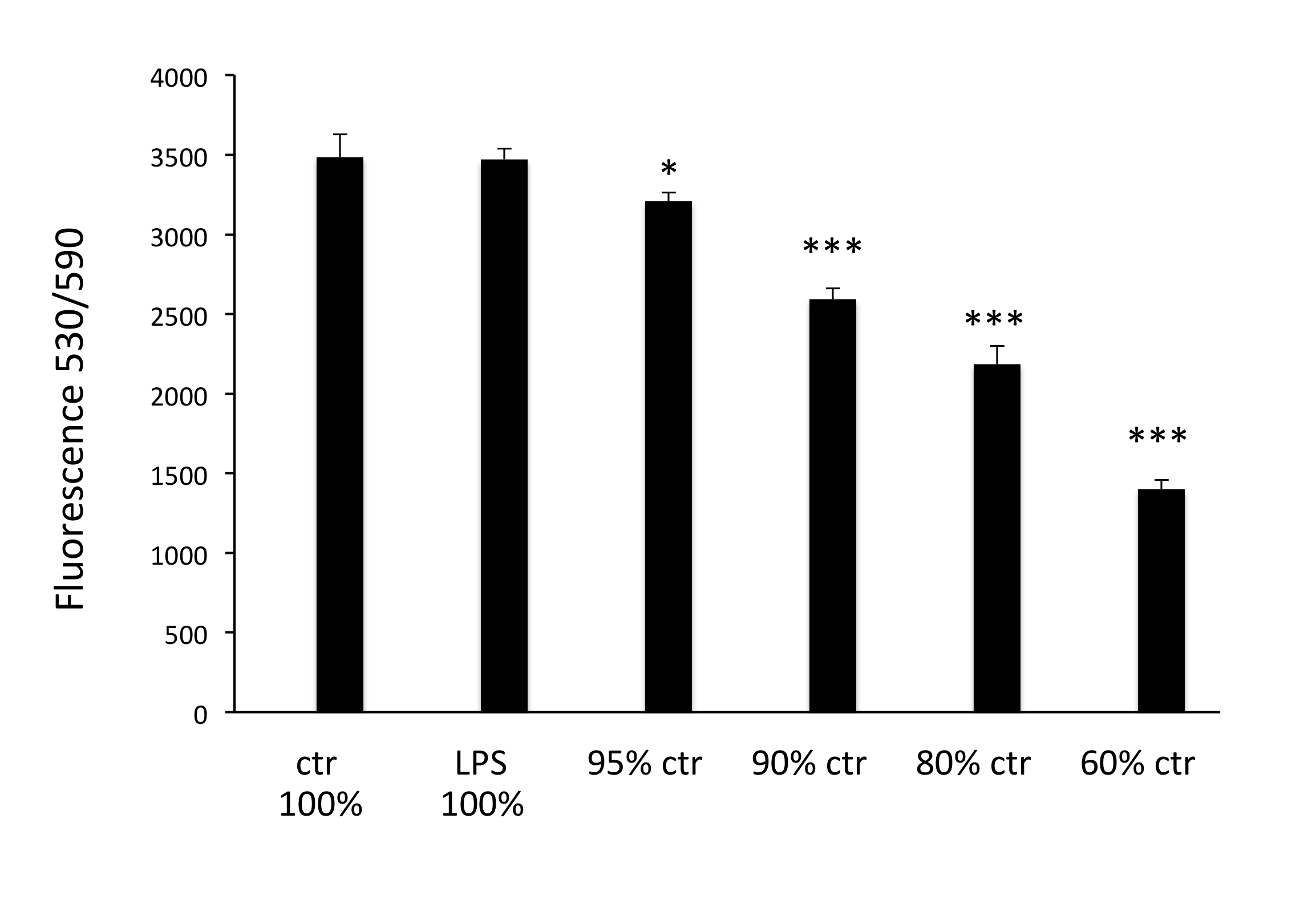

Supplement: S4 Fig — Cells were plated in 96 well plates at 25,000/well (100%) or at the indicated percentage, down to 15,000 cells/well (60%). After overnight incubation, cells were treated with LPS 100 ng/ml (LPS) or medium alone (ctr) and incubated for 24 hrs. Then CTB (20 μl /well) was added and fluorescence detected after 3h. Results are the mean ± SD of quadruplicate samples. * P < 0.05 vs ctr; ***P < 0.001 vs ctr by Student’s t-test. (TIF) [file pone.0127086.s004.tif]

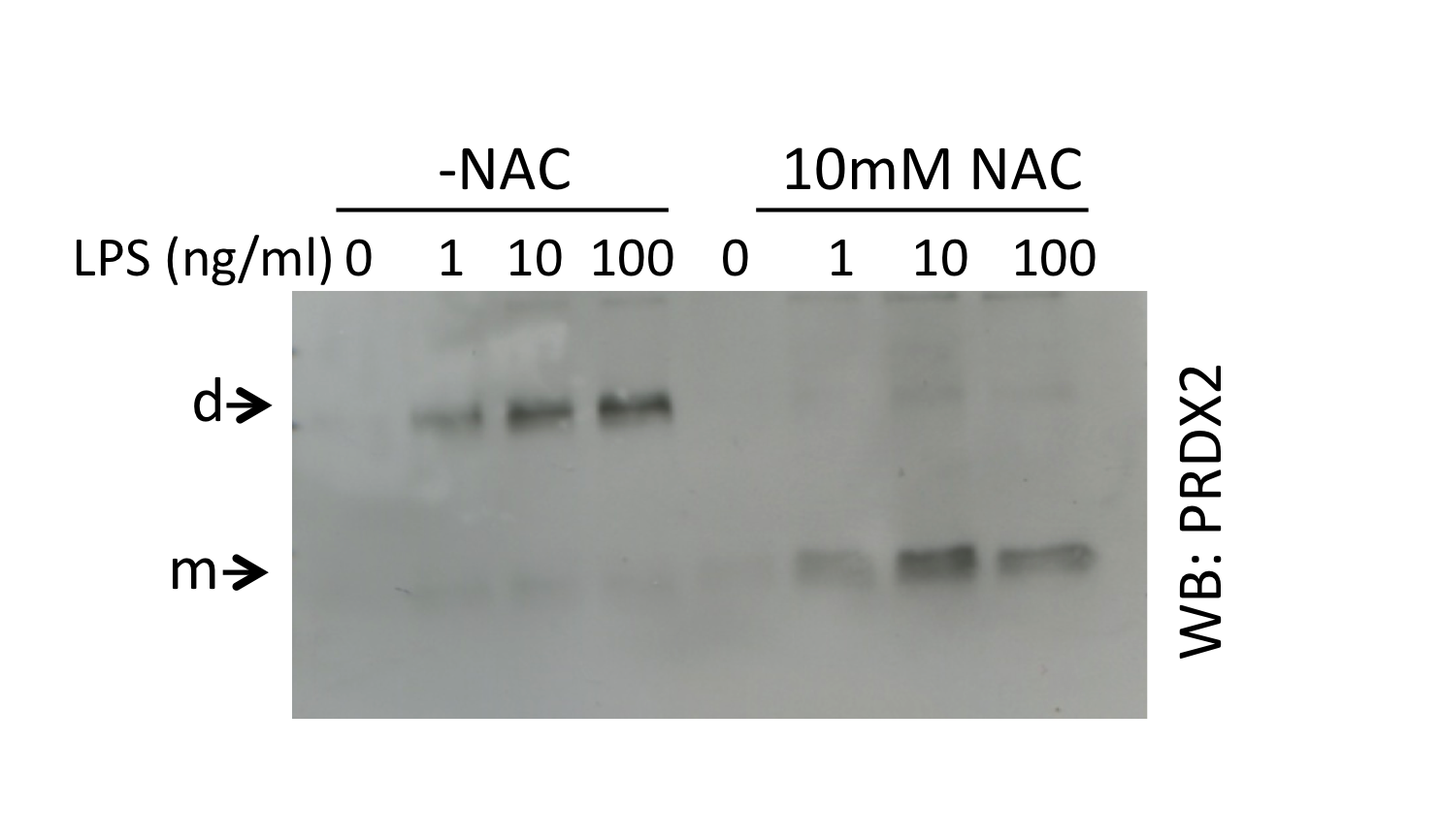

Supplement: S5 Fig — Experiment was carried out as in the legend to Fig 2, except that different concentrations of LPS were used (0–100 ng/ml), with or without 10 mM NAC present during the entire 24-h culture. (TIF) [file pone.0127086.s005.tif]
